# Recommendations for action: a community meeting in preparation for a mass-casualty opioid overdose event in Southeastern Ontario

**DOI:** 10.1186/s12919-017-0076-7

**Published:** 2017-07-18

**Authors:** Kieran Michael Moore, Nicholas Papadomanolakis-Pakis, Adrienne Hansen-Taugher, Tianxiu H. Guan, Brian Schwartz, Paula Stewart, Pamela Leece, Richard Bochenek

**Affiliations:** 1 Office of the Medical Officer of Health, KFL&A Public Health, Kingston, K7M 1V5 Canada; 2 Department of Family Medicine, Queen’s University, Kingston, K7L 5N6 Canada; 3 KFL&A Public Health, Kingston, K7M 1V5 Canada; 4 Communicable Diseases, KFL&A Public Health, Kingston, K7M 1V5 Canada; 5 Public Health and Preventive Medicine Residency Program, Queen’s University, Kingston, K7L 5N6 Canada; 6 Public Health Ontario, Toronto, M5G 1V2 Canada; 7 Dalla Lana School of Public Health, University of Toronto, Toronto, MST 1P8 Canada; 8 Office of the Medical Officer of Health, Leeds, Grenville and Lanark District Health Unit, Brockville, K6V 7A3 Canada; 9 Department of Family and Community Medicine, University of Toronto, Toronto, M5G 1V7 Canada

**Keywords:** Mass-casualty, Opioids, Public health, Surveillance, Harm reduction, Emergency preparedness, Incident management, Mass opioid overdose, Overdose surge response

## Abstract

Given the steady rise of overdose morbidity and mortality in North America, and increasing frequency of sudden clusters of non-fatal and fatal overdoses in other jurisdictions, regional preparedness plans to respond effectively to clusters of overdoses may reduce the impact of such events on the population. On the 27th of February 2017 in Kingston, Ontario, KFL&A Public Health, in collaboration with public health partners, hosted a full-day workshop involving table-top exercises and discussions for service partners on how to prepare for, respond to, and manage a mass-casualty event secondary to opioid overdose in Southeastern Ontario. The workshop assisted in identifying the various challenges faced by service partners, provided an understanding of the roles and responsibilities of partner agencies, and helped to determine next steps in preparation to address a mass opioid overdose situation at the local level. This report suggests key roles and responsibilities of partners involved in responding to a mass-casualty event secondary to opioid overdose, recommendations to address the feedback and challenges raised throughout the workshop, and a protocol to help determine when to activate an Incident Management System (IMS).

**Electronic supplementary material:**

The online version of this article (doi:10.1186/s12919-017-0076-7) contains supplementary material, which is available to authorized users.

## Background

Recent events across Ontario involving opioid overdose clusters have prompted municipalities, health system partners, and government agencies to develop plans to prepare for, respond to, and recover from a potential surge in overdoses secondary to opioids in our communities. Recently, sudden clusters of drug-related emergencies have been reported in the literature in North America and other countries, and these events threaten to overwhelm current response mechanisms and result in increased mortality [1, 2]. Rates of opioid-related morbidity and mortality in North America continue to climb in many jurisdictions. Ontario’s rate of opioid related deaths in 2015 was 5.3 per 100,000 in 2015 [3], and in the US was 10.4 in the same year [4]. British Columbia had 28.9 deaths per 100,000 involving illicit drugs in 2016, and 43.1% of the illicit drug deaths in 2015–2016 were positive for fentanyl [5].

Individuals in the population are exposed to opioids through both prescription and non-prescription sources; non-prescription opioids refer to naturally occurring opioids, semi-synthetic opioids, and synthetic opioids that may be produced illicitly [6]. Non-prescription sources of opioids are considered higher risk for overdose due to their potency, dose variability, appearance as pharmaceutical products, and potential addiction to non-opioid drugs used by opioid-naïve individuals [7]. The growing severity and scope of the opioid situation in Ontario has put the problem-solving skills of municipal, provincial, and federal leaders to the test. This issue requires planning, preparation, and collaboration among a variety of partners across the province. In developing effective and sustainable solutions to address the opioid problem, we aim to reduce the population-level harms of opioids in Ontario communities.

### Introduction: preparing for a mass-casualty event

On the 27th of February 2017, KFL&A Public Health, in partnership with the South East Local Health Integration Network (LHIN), Hastings Prince Edward Public Health, Leeds, Grenville and Lanark District Health Unit, Ottawa Public Health, and Public Health Ontario, hosted a full-day workshop involving table-top exercises and discussions on how to prepare for, respond to, and manage a mass-casualty event secondary to opioid overdose in South Eastern Ontario. The workshop brought together 93 officials representing emergency first responders, acute care and health service providers, community, mental health and addictions services, and local, federal, provincial and territorial (FPT) public health partners. The workshop intended to identify the various challenges faced by service partners, clarify roles and responsibilities of partner agencies, and determine next steps to address a mass-casualty opioid overdose situation at the local level.

This report is the result of summarized discussions, worksheets, and feedback received throughout the day-long workshop. It suggests key roles and responsibilities of partners involved in responding to a mass-casualty event secondary to opioid overdose, and a protocol to help determine when to activate an Incident Management System (IMS). These findings can be adapted in other communities across North America similarly affected by opioid-related harms in the population to inform emergency preparedness plans as part of comprehensive planning to reduce morbidity and mortality due to opioids.

## Methods

The Mass Opioid Overdose Workshop was designed utilizing a health emergency management simulation exercise framework [8]. Ninety three participants attended the workshop representing emergency first responders, acute care and health service providers, community health, mental health and addictions services, and local, federal, provincial and territorial (FPT) public health partners. The workshop consisted of a combination of three simulation exercises and facilitated tabletop discussions. The selected scenarios were based on factual events [1, 9] with varying levels of risk. A facilitated discussion followed each exercise to determine what worked well, challenges in the response and how they could be addressed, scenario outcomes, and to identify issues raised in the exercise. Information was collected by transcribing discussions and summarizing worksheets and debrief notes. Recommendations were developed based on summarized information and best-practices. A voluntary and anonymous workshop evaluation survey was disseminated to participants to collect feedback on their overall experience.

### Key roles and responsibilities

This section outlines the key roles and responsibilities in managing a mass-casualty opioid overdose event as identified by participants in the workshop. The following list is intended for partners to understand each other’s key roles in the critical first few hours of a mass opioid casualty event, the on-going management of the situation, and recovery phase. The list includes the most relevant roles and responsibilities for emergency first responders, local and regional health services, and FPT partners.

### Local & regional health services

#### Public health

##### Surveillance

- Determine the epidemiological triggers for an alert through analysis of surveillance data from partners.
- Create a community task force, with terms of reference, for sharing situational awareness and surveillance data, on an ongoing regular basis.

##### Communication

- Communicate dangers of exposure to opioids to community partners, people who use drugs, and the public through a preplanned multi-component strategy.
- Communicate the confirmed or suspected presence of high-risk opioids to community partners and the public.
- In addition to monthly reporting and information sharing by the task force, share epidemiological and other relevant information with acute care and community service providers.

##### Harm reduction

- Assist with the distribution of naloxone in the community and support an urgent response to increase the availability of naloxone to community members at-risk of experiencing or witnessing an opioid-related health emergency.
- Connect with community health, mental health and addictions partners to ensure people who use drugs and those close to them (e.g., family, friends, service providers) have the proper training on harm reduction.
- Provide needle exchange and education programs.

#### Acute care

##### Manage opioid overdose cases

- Provide emergent healthcare, prepare for surges in emergency department visits, prepare code orange responses [10], ensure the availability of intensive care unit beds and naloxone, provide naloxone kits to overdose patients upon discharge, refer patients to community treatment and harm reduction services or other appropriate services post-discharge.

##### Communication

- Rapidly communicate suspected opioid overdose surges to local, regional and provincial public health authorities and relevant agencies as part of the hospital disaster plan.

##### Surveillance

- Count probable and confirmed cases (see Additional file 1), send toxicology on initial cases, and document toxicology results.
- Assess patient medical history and identify possible links between cases.

#### Community health, mental health and addictions services

##### Harm reduction

- Provide outreach services to high-risk groups and settings to assist with risk communication and provision of harm reduction services.
- Provide naloxone kits, training, and refills for clients.
- Ensure availability of supervised consumption sites (if warranted) and effective needle exchange programs.
- Provide opioid agonist therapy and mental health and addictions services.

##### Education

- Disseminate information and notify staff and regular clients of the dangers associated with opioid use.

##### Communication

- Communicate to staff members, community partners, and people who use drugs on the highly toxic opioids entering the drug supply chain within the community.
- Utilize a community communication distribution list and protocol to reach clients and community partners.
- Collect and collate information from clients and determine whether the high-risk opioids are from a common source.

### Emergency first responders

#### Paramedic services

##### Provide primary and advanced paramedicine care

- Respond to calls for service, provide emergency care to victims in the field and en route to acute care centres, and prepare for a response to a surge in call volume and naloxone requirements.

##### Connect with para-medicine partners

- Upon recognition of an increase in opioid overdoses (above baseline levels), provide immediate situational awareness to emergency medical services dispatch centres and partner paramedic services. Request assistance and resources from partner paramedic services as needed.

#### Police services

##### Communication

- Police were identified by many participants as the communication lead and therefore should establish incident command and manage communications between first responders, emergency department, public health, and partners utilizing an IMS structure.
- Provide a joint communications release as needed to the public via social media, press conference etc.

##### Enforcement

- Initiate an investigation to determine the source of high-risk opioids involved.

##### Support paramedic services

- Assist with scene management.

#### Fire services

##### Support emergency first-response partners

- Support paramedic services by providing in-field first-aid (including naloxone administration if trained and permitted by the local jurisdiction) prior to paramedic arrival. This is of importance when paramedics experience hospital off-load delays or when overwhelmed by demand for service.
- Provide Hazardous Materials (HazMat) services and support if required.

### Federal, provincial and territorial partners

#### Provincial and territorial governments

##### Support

- Support regions and municipalities with coordination, material support and leadership if required throughout a mass-casualty opioid overdose event through existing emergency structures and processes, including IMS.
- Develop provincial policy to facilitate local level action.

##### Surveillance

- Conduct surveillance with local partners.

##### Communication

- Notify regional health authorities of a response to an increase in opioid-related overdose emergencies.
- Provide a public health advisory to the public and health professionals.
- Assist with debrief for lessons learned during the recovery phase of emergency management.

#### Federal government

##### Communication

- Assist with debriefing and sharing with other provinces and territories the lessons learned following multi-provincial/territorial response to an opioid-related emergency.

##### Policy

- Inform provincial policy direction.
- Develop and implement evidence-based policy across Canada.
- Access to emergency stockpiles of resources if required.

##### First nations health services

- Provide on-reserve emergency care services in remote and isolated areas.

##### Research

- Review scientific evidence and share what is happening across the country.

### Recommendations

The following recommendations are suggested by KFL&A Public Health to address the feedback and challenges raised throughout the workshop regarding the response to, and prevention of, a mass-casualty opioid overdose event. In total, 15 recommendations emanated from discussion at the workshop. Some of the major challenges that were identified by participants included: determining the triggers for an urgent community response, communicating and coordinating between the various partners, responding to emergencies in rural communities, resource strain, privacy implications for investigative purposes, and after-action evaluation for continuous quality improvement and lessons learned.

These recommendations are intended for emergency first response agencies, public health, healthcare providers, community health, mental health and addictions services, and FPT government agencies.

i. Recommendations for emergency first responders, public health, acute care, community health, mental health and addictions services, and municipal, federal, provincial and territorial (FPT) partners.

1. *Establish or join a local task force to address the opioid overdose problem. Include officials from: public health, acute care, community health, mental health and addiction services, emergency first response agencies, community emergency management coordinators, post-secondary education institutions, school boards, and the regional coroner’s office.*

Several participants from the workshop felt their agencies were not receiving up-to-date information on opioid-related trends and initiatives.

A local task force will allow municipalities and local partners/service providers to maintain regular communication and share new and emerging trends from surveillance data and analysis. The task force could also discuss and develop solutions to address opioid overdose-related issues in their communities and to prevent further harm from occurring.

Currently, local public health in Kingston leads a task force comprised of three local public health agencies in the region, municipal police services, Ontario Provincial Police, Kingston Community Health Centre-Street Health, hospital emergency departments, three paramedic services, County Fire Service coordinator, and the regional coroner’s office (see Additional file 2 for draft terms of reference). This task force meets monthly and has been successful in maintaining regular communication and sharing situational awareness, timely updates on surveillance data, and initiatives for harm reduction, education, and enforcement measures around the supply of opioids in the region.

2. *Develop a mass-casualty opioid overdose response plan in anticipation of a surge within the community. The plan, developed by local public health agencies, hospitals, regional health authorities, emergency first responders, community health, and mental health and addictions services could describe:*
  - *The various roles and responsibilities involved in the response of each sector.*
  - *Plans to share information including a list of key contacts, and the point or trigger at which specific agencies and officials at various levels of government should be contacted and informed of the surge event.*
  - *Plans to acquire additional support and resources should they be overwhelmed (*i.e. *pre-hospital response, treatment supplies, patient transfers).*

Coordination and planning was identified by all participants as integral for responding to a mass-casualty opioid overdose event. Participants expressed concern over coordination, and not knowing who should be contacted, and at what point in the response they should be contacted.

Interagency collaboration is required to coordinate communications and resources, and to develop a plan to meet short and long-term objectives. Emergency preparedness involves awareness and understanding of the roles and responsibilities of your and partner agencies. Developing a response plan will provide an opportunity to organize an effective, efficient, and coordinated response to a surge in opioid-related overdoses.

3. *Officials at the local community level collaborate with federal, provincial and territorial (FPT) partners to debrief following a response and disseminate lessons learned to partners within and outside of the province.*

It was noted by FPT partners that after-action debriefing is often overlooked.

Each organization playing a role in a response effort to manage an increase in opioid-related harms should evaluate its actions and the associated outcomes to ensure continuous quality improvement and identify solutions to improve future emergency responses. FPT governments may assist with debriefing and developing and disseminating the lessons learned on what worked and what aspects require improvement.

4. *Increase public awareness on opioid-related harms and educate communities on how they can contribute to reducing harms. The funding and resources necessary to ensure information dissemination should be secured.*

The workshop demonstrated the need to share information with the public regarding the dangers of opioid use or unintentional exposure to opioids, and supports available in the community.

All organizations and service providers should have a role in a public awareness campaign utilizing various methods of information sharing including pamphlets, news media, social media, presentations, and town hall meetings. Communication to the public should be conducted in collaboration with the local public health agency to ensure information being received by the community is evidence-informed and up-to-date.

5. *Local public health agencies coordinate with other community partners to provide educational sessions, and assist in disseminating informational material to frontline community partners and the public.*

Participants identified a lack of education and training for employees in their respective agencies.

Education and training for frontline workers can minimize the harms associated with opioid use. Frontline agencies, including emergency first responders, and community, mental health and addictions services, in collaboration with the local public health agency, could provide adequate training and education to their employees about overdose prevention, recognition, and emergency response among their clients. Additionally, training could include the dangers of occupational exposure to high-risk opioids including fentanyl and its analogues, and the proper handling procedures for these substances. Education and training will aid in understanding the importance and severity of opioid-related harms and help to provide safer, more effective services to the public.

6. *Develop a provincial standardised descriptive diagnostic coding scheme to improve surveillance for opioid overdose events and communication amongst healthcare providers.*

Participants employed in health-related sectors expressed a need for a common provincial language or diagnostic codes to describe opioid overdose events. When information is received from other healthcare providers, there is confusion surrounding the reason for patient visits (i.e. respiratory arrest/vital signs absent/overdose/substance misuse). The absence of standardized language negatively impacts the sensitivity and specificity of surveillance systems and the ability to detect a signal or change in the baseline data.

The development of a standardized diagnostic coding scheme for opioid-related health emergencies could improve surveillance capabilities and communication between healthcare providers and agencies, and increase the efficiency of a community response when managing surges in opioid overdoses.

ii. Recommendations for public health.

7. Local public health agencies develop and deliver a communication strategy in collaboration with schools which includes information for teachers, parents, and students.

Discussion amongst participants concerning recent opioid overdose deaths in Ottawa, Ontario revealed the need for a strategy to communicate the dangers of opioid exposure to youth, parents, and teachers.

A recent fentanyl-related death involving a young teenager has demonstrated the young age at which individuals can be exposed to opioids. In partnership with school boards, public health agencies should develop an effective communication strategy to: a) educate and increase awareness among youth about the dangers of exposure to opioids, b) reduce stigma surrounding drug use, addiction, and seeking treatment, and c) educate youth, parents, and teachers on how they can prepare themselves to assist a potential overdose victim.

8. *Local public health agencies in the province could conduct surveillance of hospital emergency department visits and admissions, paramedic services, coroner, and naloxone distribution/usage data to detect surges in baseline opioid overdoses.*

Real-time situational awareness of overdose surveillance data was identified by participants as a key mechanism to be able to respond to opioid overdose surge events.

KFL&A Public Health conducts syndromic surveillance on a real-time basis as data is forwarded from local emergency departments to the public health agency electronically, via the Acute Care Enhanced Surveillance System (ACES). In turn, KFL&A Public Health has the ability to detect surges in baseline overdose data and trigger an alert to health service providers and community partners of a potential need for an urgent community response.

iii. Recommendations for public health, community health, mental health and addictions services, and federal, provincial and territorial partners.

9. *Increase the distribution of naloxone kits to community health, mental health and addictions services providers, people who use drugs and their friends and families, and students in high-risk areas including rural First Nations communities. Increase the number of pharmacy partners distributing naloxone kits.*

Provision of a naloxone supply and enhanced medical care to rural First Nations communities were identified as challenges in responding to a mass-casualty opioid overdose incident in this jurisdiction.

The high rates of substance use in First Nations communities [11–13] coupled with lengthy emergency response times and limited health services in rural areas illustrates the need for naloxone kits and training to be made readily available to individuals in high-risk communities.

10. *Facilitate and increase the availability of treatment and counselling for substance use disorders, needle exchange and safe disposal sites, and naloxone kits for people at-risk of experiencing or witnessing an opioid overdose.*

Participants felt that efforts to manage opioid overdoses were largely focused on reactive approaches.

There is a need for preventative and post-overdose care approaches through improved access to treatment and counselling for substance use disorders, social supports, and harm reduction efforts. Opioid agonist treatments significantly reduce mortality among people dependent on opioids [14]. Further, effective harm reduction strategies will minimize the harms associated with substance use and prevent the spread of infectious disease by eliminating the need to share needles.

iv. Recommendation for police services and federal, provincial and territorial partners.

11. *In collaboration with judicial system partners, target the sources of illicit opioids, and develop a plan to divert individuals with substance use disorders away from the criminal justice system to seek appropriate treatment and counselling.*

There was concern among participants that too much police involvement would lead to an enforcement approach to a public health issue. Police also identified the inability to retrieve information from patients in hospital as a challenge to fulfilling their investigative role effectively.

An enforcement approach to a public health issue does not address the root cause of the problem. Police, in collaboration with partners of the judicial system, could focus their efforts and resources on reducing the supply of illicit opioids, and divert individuals with substance use disorders away from the criminal justice system towards appropriate treatment. If individuals are afraid of criminal charges for using substances, they may be reluctant to report or provide information regarding an overdose that may be crucial to providing emergency medical attention and initiating an investigation. As such, the Government of Canada has recently amended the *Controlled Drugs and Substances Act* with the *Good Samaritan Drug Overdose Act* [15] to provide immunity from simple possession for anyone that seeks emergency assistance for themselves or another person suffering from an overdose.

v. Recommendation for paramedic services and federal, provincial and territorial partners.

12. *Increase the supply of naloxone to paramedic services.*

Paramedic services participants identified a limited naloxone supply as a challenge to providing pre-hospital care during an opioid overdose surge event.

Currently paramedic service crews only carry enough naloxone for two patients. It is possible that a single patient may require multiple doses of naloxone to reverse the effects of an opioid overdose. Provincial and territorial governments could work with paramedic services to ensure the appropriate amount of naloxone is available and stocked within each service.

vi. Recommendations for federal, provincial and territorial partners.

13. *Federal, provincial and territorial governments support strategies to respond to a mass-casualty opioid overdose emergency in rural communities, including increasing the supply of naloxone.*

Participants identified that responding to rural communities would be difficult and potentially have lengthy response times.

The impact of opioid use is considerable amongst vulnerable populations including youth, seniors, First Nations, and those living in poverty [11]. Strategies to reduce the time it would take to respond to a potential increase in opioid-related health emergencies in a rural community, including patient transport to hospital, should be developed and implemented.

14. *Develop a streamlined system for toxicology testing where an opioid overdose has been suspected.*

Acute care, public health, and police participants expressed concern about the length of time experienced to receive results of toxicology testing in suspected opioid overdose cases.

Currently, it could take up to several weeks to receive toxicology results. In the case of an opioid overdose surge, timely results would allow healthcare practitioners to confirm the diagnosis, speed-up the investigation, and help prevent further overdoses. Local task forces should create an opioid testing protocol in advance that would result in the most rapid detection of the responsible opioid(s).

15. *Equip police and fire services in high-risk communities to administer naloxone to potential overdose victims.*

This recommendation was mentioned in discussion as a future consideration for police officers and firefighters to carry naloxone in communities with high rates of overdoses.

The *Good Samaritan Act* [16] provides legal protection for anyone providing reasonable care to an individual in an emergency. Fire services often have shorter response times than paramedic services, and police services often patrol communities where drug use is visible in the streets. In these circumstances, the ability for firefighters or police officers to administer naloxone may, in some cases, be the difference between life and death.

### Opioid overdose surge response plan

**Fig. 1:**
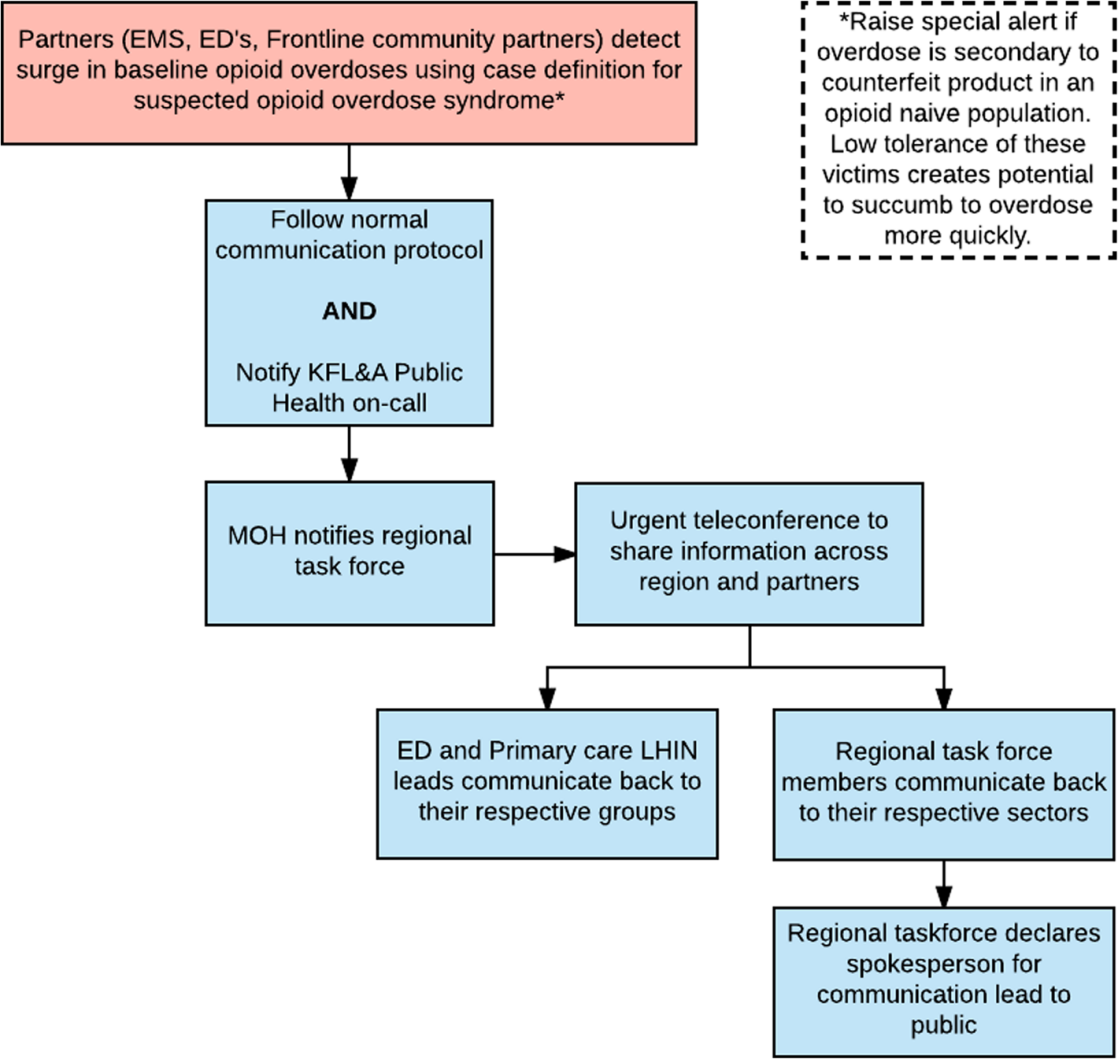
Communication pathway for notifying the health system during an opioid overdose surge

Regional surveillance partners, such as acute care, paramedic, fire and police services, should have knowledge of the baseline level of opioid overdoses within their communities or service provision jurisdiction. Upon recognizing a deviation from this baseline, notification can be made to public health. Fig. 1 illustrates a proposed notification and communication pathway utilizing the Joint Agency Group on Drugs of Abuse (regional task force). A case definition of suspected opioid overdose syndrome is proposed (see Additional file 1).

**Fig. 2:**
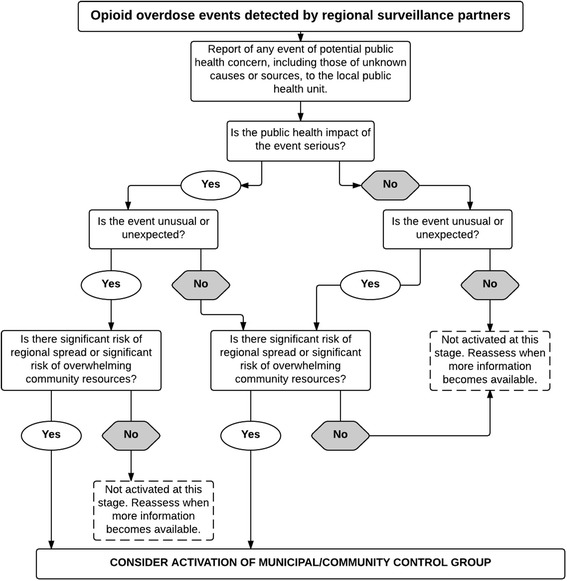
Decision instrument to activate municipal/community control group and IMS. Adapted from the WHO (2008) International Health Regulations (2005) 2nd ed. [17]

There was consensus from participants that the municipal/community control group (MCG/CCG) may be activated in response to a significant increase in opioid overdoses. Although an IMS model is preferred, exact details regarding a framework were not discussed. Fig. 2 describes a decision-making tool to assist MCG/CCG members in determining when to activate in response to a significant opioid overdose surge event.

**Fig. 3:**
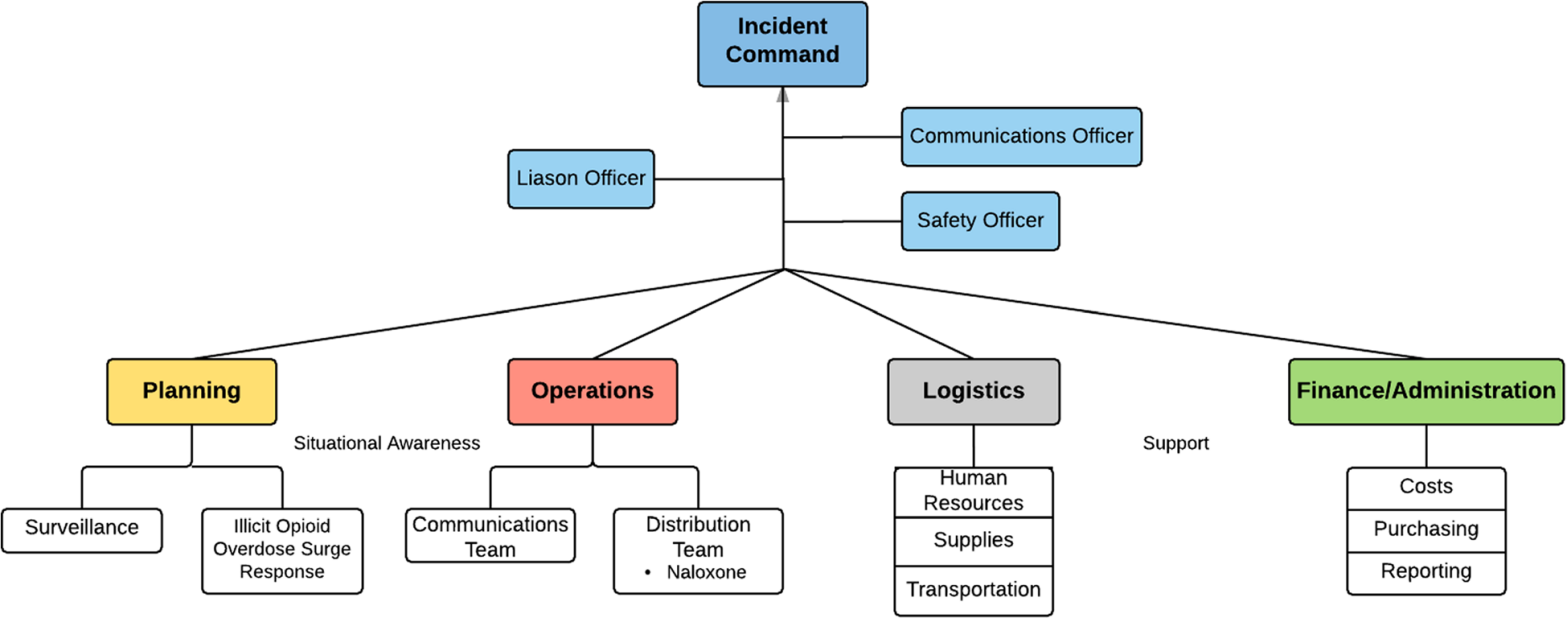
Public health opioid overdose surge response utilizing a standard incident management structure

An effective response requires key strategies be operationalized in an effective and efficient manner. The following is a proposed public health IMS framework for use during opioid overdose surge events (Fig. 3). It is important to note that IMS is not a light switch; it is possible to initiate IMS protocol early, as one individual may perform multiple IMS functions, and the level of response is scalable.

**Fig. 4:**
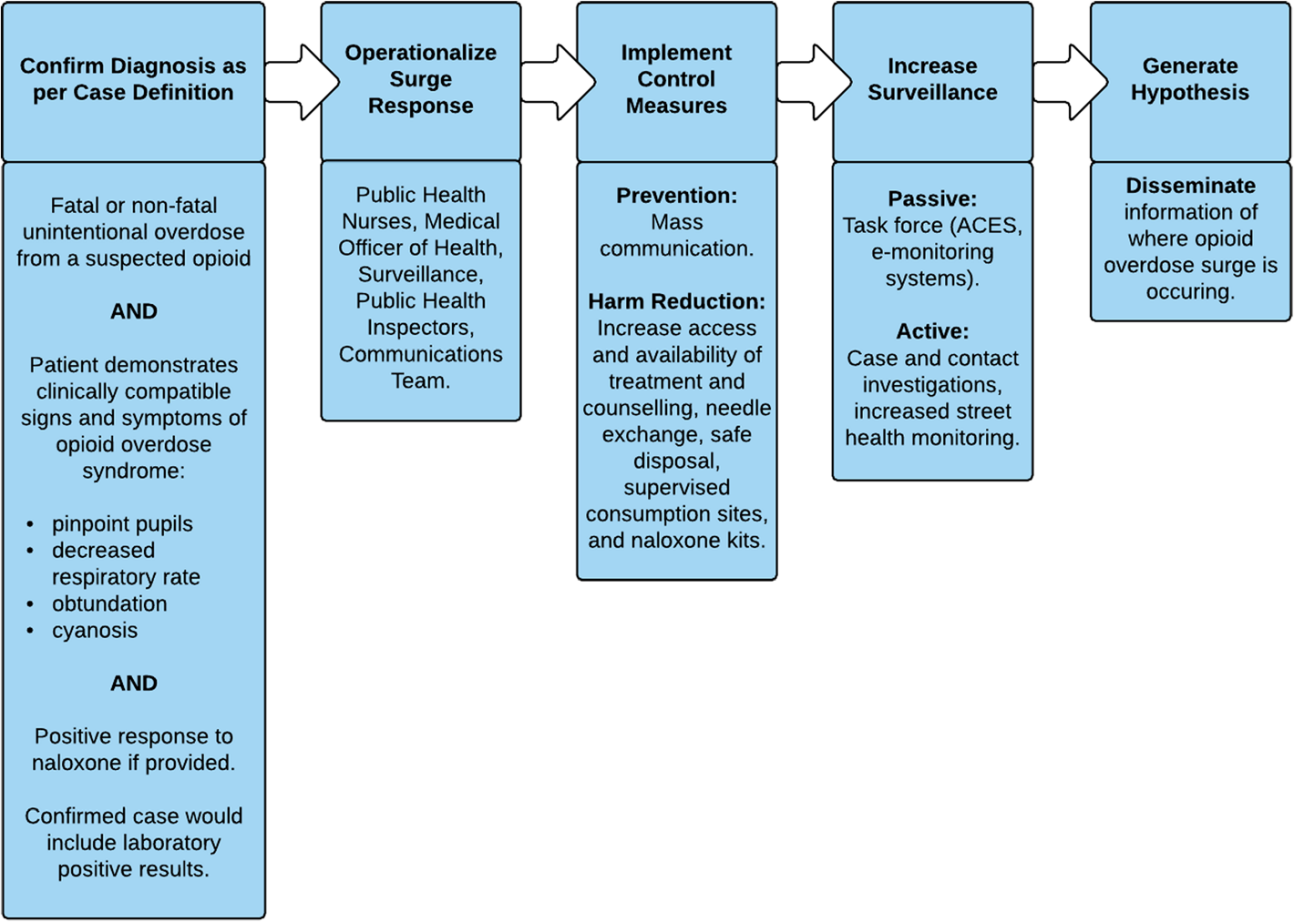
Public health opioid overdose surge response pathway

The *Public Health Overdose Surge Response Pathway* (Fig. 4) outlines the processes needed to prepare for, respond to, and recover from an unusual or unexpected public health threat. The pathway is activated within the context of a community-based response to opioid overdose surge events.

## Conclusion

The growing severity and scope of the opioid situation in Ontario has put the problem-solving skills of municipal, provincial, and federal leaders to the test. The Mass Opioid Overdose Workshop hosted by KFL&A Public Health demonstrated the complex nature of the planning and preparation required to effectively respond to a mass-casualty opioid overdose event. In developing effective and sustainable solutions to address the issue of opioid overdoses, we aim to reduce the population-level harms of opioids in Ontario communities. Our learning through the emergency planning process may be valuable to other jurisdictions facing similar morbidity and mortality related to opioids.

## Additional files

Additional file 1: Case definition of suspected opioid overdose syndrome. (DOCX 44 kb)

Additional file 2: Draft Terms of Reference: Joint Agency Group on Drugs of Abuse. (DOCX 101 kb)

## Abbreviations

ACES: Acute care enhanced surveillance system
CCG: Community control group
FPT: Federal, provincial and territorial
HazMat: Hazardous materials
IMS: Incident management system
LHIN: Local health integration network
MCG: Municipal control group
