# Supplementary material for: Recommendations for action: a community meeting in preparation for a mass-casualty opioid overdose event in Southeastern Ontario
Source: BMC Proc. 2017 Jul 18;11(Suppl 7):8. doi: 10.1186/s12919-017-0076-7 (PMC5547449; doi:10.1186/s12919-017-0076-7)
Supplement: Supplementary file 1 — Case definition of suspected opioid overdose syndrome. (DOCX 44 kb) [file 12919_2017_76_MOESM1_ESM.docx]

Additional file 1

**Case Definition: Opioid Overdose Syndrome**

1. **Probable**

Fatal or non-fatal unintentional overdose from a suspected opioid

**AND**

Patient demonstrates clinically compatible signs and symptoms of opioid overdose syndrome:

- Pinpoint pupils
- Decreased respiratory rate
- Obtundation
- Cyanosis

**AND**

Positive response to naloxone (if provided).

1. **Confirmed**

All the above **plus** laboratory confirmation of opiate exposure.
