# Supplementary material for: Recommendations for action: a community meeting in preparation for a mass-casualty opioid overdose event in Southeastern Ontario
Source: BMC Proc. 2017 Jul 18;11(Suppl 7):8. doi: 10.1186/s12919-017-0076-7 (PMC5547449; doi:10.1186/s12919-017-0076-7)
Supplement: Supplementary file 2 — Draft Terms of Reference: Joint Agency Group on Drugs of Abuse. (DOCX 101 kb) [file 12919_2017_76_MOESM2_ESM.docx]

Additional file 2

**Draft Terms of Reference: Joint Agency Group on Drugs of Abuse**

**1. Purpose**

To provide a forum for effective communication, coordination, and collaboration between agencies on narcotic and drugs of abuse control issues.

**2. Activities**

a. Provide awareness and share information of importance to the member agencies and other relevant enforcement agencies as needed.

b. Address and make recommendations on issues affecting narcotic/drugs of abuse control.

c. Co-ordinate enforcement and policy activities as needed.

**3. Membership**

Membership is on a voluntary basis and will be representative of all member agencies.

Kingston, Frontenac and Lennox & Addington Public Health, Hastings and Prince Edward Counties Health Unit, Leeds, Grenville & Lanark District Health Unit, Ontario Provincial Police, Royal Canadian Mounted Police Customs & Excise Unit, Kingston Police Force, Canada Border Services Agency Lab, and Ministry of Revenue -Special Investigations Branch, Regional Coroner, EMS/Fire, Street Health, Needle Exchange program, and others.

**4. Roles**

**4.1 Chair**

A representative from KFL&A Public Health shall act as Chair.

**4.2 Recorder**

The recorder will be assigned on an alphabetical rotating basis according to last name.

**5. Decision-making**

Decisions are made by consensus of the committee. Where no agreement is reached, that matter

may be decided by the Chair or referred for future consideration by the committee.

**6. Meetings**

The committee will meet quarterly, or more frequently as needed.

**7. Documentation**

The events of each meeting will be recorded and distributed to all committee members. Relevant documents will be appended for reporting purposes. A copy of the minutes will be kept on file by the Chair.
